# Silencing of lncRNA PKIA-AS1 Attenuates Spinal Nerve Ligation-Induced Neuropathic Pain Through Epigenetic Downregulation of CDK6 Expression

**DOI:** 10.3389/fncel.2019.00050

**Published:** 2019-02-20

**Authors:** Jian-Zhong Hu, Zi-Jie Rong, Miao Li, Ping Li, Li-Yuan Jiang, Zi-Xiang Luo, Chun-Yue Duan, Yong Cao, Hong-Bin Lu

**Affiliations:** ^1^Department of Spine Surgery, Xiangya Hospital, Central South University, Changsha, China; ^2^Key Laboratory of Organ Injury, Aging and Regenerative Medicine of Hunan Province, Xiangya Hospital, Central South University, Changsha, China; ^3^Department of Obstetrics, Xiangya Hospital, Central South University, Changsha, China; ^4^Department of Sports Medicine, Xiangya Hospital, Central South University, Changsha, China

**Keywords:** neuropathic pain, long non-coding RNA, spinal cord injury, neuroinflammation, CDK6

## Abstract

Neuropathic pain (NP) is among the most intractable comorbidities of spinal cord injury. Dysregulation of non-coding RNAs has also been implicated in the development of neuropathic pain. Here, we identified a novel lncRNA, PKIA-AS1, by using lncRNA array analysis in spinal cord tissue of spinal nerve ligation (SNL) model rats, and investigated the role of PKIA-AS1 in SNL-mediated neuropathic pain. We observed that PKIA-AS1 was significantly upregulated in SNL model rats and that PKIA-AS1 knockdown attenuated neuropathic pain progression. Alternatively, overexpression of PKIA-AS1 was sufficient to induce neuropathic pain-like symptoms in uninjured rats. We also found that PKIA-AS1 mediated SNL-induced neuropathic pain by directly regulating the expression and function of CDK6, which is essential for the initiation and maintenance of neuroinflammation and neuropathic pain. Therefore, our study identifies PKIA-AS1 as a novel therapeutic target for neuroinflammation related neuropathic pain.

## Introduction

Neuropathic pain (NP) is among the most intractable comorbidities of spinal cord injury (SCI). It can manifest as multiple forms of chronic pain in areas where skin sensation is lost below the injury plane (Dones and Levi, 2018). The incidence of NP in patients with SCI is increasing annually (Wayne, 2018). There are currently no curative therapies for SCI-associated NP, and clinical management is limited to symptomatic treatment. Further, treatment efficacy is often not ideal, which seriously affects patient quality of life. Indeed, many patients experience depression and anxiety, develop drug addictions, and even contemplate or attempt suicide.

The release of inflammatory cytokines from activated astrocytes and microglia is strongly implicated in the development of neuropathic pain (McKelvey et al., 2015). As inhibition of neuroinflammation by inactivation of astrocytes and microglia can ameliorate pain-related behavior (Walters, 2014). In addition, genetic deletion of cell cycle-related proteins such as cyclin-dependent kinases (CDKs) has also been shown to reverse pain-related behavior (Gwak and Hulsebosch, 2011). Nociception is an integral component of host defense, but prolonged nociceptive input can drive central neuroinflammation and induce neuroplastic changes within pain pathways (Gwak et al., 2012). It has been suggested that spinal injury can activate nociceptor-mediated host defense responses via neuroinflammatory signaling to produce chronic pain (Coraggio et al., 2018).

Non-coding RNAs (ncRNAs), including microRNAs and long non-coding RNAs (lncRNAs), contribute to diverse biological processes by regulating the expression of mRNA targets at the post-transcriptional level (Bali and Kuner, 2014; Zhou et al., 2017) by targeting several pain pathways (Tan et al., 2015; Plassais et al., 2016; Shao et al., 2018). Dysregulation of non-coding RNAs has also been implicated in the development of neuropathic pain (Liu et al., 2017; Vieira et al., 2018). For example, lncRNA BC168687 is associated with diabetic neuropathic pain (Liu et al., 2018), while lncRNA MRAK009713, NEAT1, and XIST can regulate chronic constriction injury-induced neuropathic pain (Li et al., 2017; Xia et al., 2018; Yan et al., 2018).

Here, we identified a novel lncRNA, PKIA-AS1, by using lncRNA array analysis in spinal cord tissue of spinal nerve ligation (SNL) model rats, and investigated the role of PKIA-AS1 in SNL-mediated neuropathic pain. We observed that PKIA-AS1 was significantly upregulated in SNL model rats and that PKIA-AS1 knockdown attenuated neuropathic pain progression. Alternatively, overexpression of PKIA-AS1 was sufficient to induce neuropathic pain-like symptoms in uninjured rats. We also found that PKIA-AS1 mediated SNL-induced neuropathic pain by directly regulating the expression and function of CDK6, which is essential for the initiation and maintenance of neuroinflammation and neuropathic pain. Therefore, our study identifies PKIA-AS1 as a novel therapeutic target for neuroinflammation related neuropathic pain.

## Materials and Methods

### Animal Model

Male Sprague-Dawley (SD) rats (∼200 g) were obtained from the Experimental Animal Central of Central South University. The animal experiments were approved by the Animal Care and Use Committee of Central South University. Spinal nerve ligation (SNL)-induced neuropathic pain model was established as previous described (Zhang et al., 2013). Briefly, following anesthetized with 1% sodium pentobarbital (40 mg/kg i.p.), the fifth lumbar (L5) spinal nerves were isolated and ligated tightly with 6–0 silk suture. For sham-operated mice, rats received identical surgical procedure without ligation. Mechanical withdrawal threshold (MWT) and thermal withdrawal latency (TWL) were used to validate the SNL model.

### Animal Infection

To test the effect of lentivirus shPKIA-AS1 (Lv-shPKIA-AS1, sequence: CCGGGACGACCCTGACCATGGTCGCGATATCTCGAGATATTTGAGGTCAGGGTCGTCAATTTG, Sigma Aldrich), SNL rats were received intrathecal injections of either Lv-shPKIA-AS1 (MOI = 100) or the lentivirus with scramble sequence on the third day after surgery.

To assess the effect of PKIA-AS1 overexpression, rats were received intrathecal injections of either Lv-PKIA-AS1 [multiplicity of infection (MOI) = 100] or the lentivirus with scramble sequence on the fifth day of observation.

To assess the effect of CDK6 overexpression, rats were received intrathecal injections of either Lv-CDK6 (MOI = 100) or the lentivirus with scramble sequence on the third day after surgery. Intrathecal injection process was performed as previously described (Zhang et al., 2013). Briefly, rats were placed in a prone position after anesthesia. After creating a small opening at the intervertebral space between the L4–L6 vertebrae, a sterile PE10 intrathecal catheter was inserted through the opening into the lumbar enlargement. After catheterization, all rats were allowed to recover for two days before being used in other experiments.

### Pain Threshold Assessment

Mechanical allodynia was evaluated by paw withdrawal threshold (PWT) using von Frey filaments. Briefly, rats were put into a transparent plastic box (22 cm × 12 cm × 22 cm). The box has a metal mesh floor. The calibrated von Frey filaments (IITC, Woodland Hills, CA, United States) were used to make pressure on the plantar surface of rat hind paw. The investigators recorded the size of the filaments when paw withdrawal. Paw withdrawal latency (PWL) was used to evaluated thermal hyperalgesia by the Plantar Test Instrument (Hargreave’s Method). The hind paws were tested alternately at 5-min intervals. The investigators recorded the duration between stimuli and paw withdrawal. The cut-off time was at 30 s. All tests were performed blindly.

### LncRNA Microarray Analysis

On the 15th day after spinal nerve ligation (SNL) or sham operation, rats were anesthetized with pentobarbital sodium (150 mg/kg IV) and their spinal cord tissues (L5) were removed and frozen by liquid nitrogen immediately. RNeasy Mini Kit (Qiagen, GmBH, Hilden, Germany) was used to extract total RNA from spinal cord tissues according to the manufacturer’s instructions. NanoDrop 2000 spectrophotometer was used to quantify purified total RNA. To analyze lncRNA expression profiles (Arraystar Rat LncRNA Expression Array V2.0), the purified total RNA was sent to Novogene Co. Ltd. (Beijing, China). Differentially expressed lncRNAs were identified through fold change (>2) and *P*-value (<0.05).

### RNA Extraction and qRT-PCR

TRIzol reagent (Invitrogen) was used to extract total RNA from cells or tissues. Maxima First Strand cDNA Synthesis kit (cat no. K1642; Thermo Fisher Scientific, Inc.) was used for reverse transcription according to the manufacturer’s protocol. Quantitative PCR amplification was performed with an CFX96 Touch^TM^ Deep Well Real-Time PCR Detection System (Bio-Rad, Hercules, CA, United States). Expression of lncRNAs, PKIA-AS1, IL-1β, IL-6, IL-12, TNFα, and CDK-6 was detected via UltraSYBR Mixture (cat. no. CW2602; CWBio) according to the manufacturer’s protocol. QPCR was performed at the condition: 95.0°C for 3 min, and 39 circles of 95.0°C for 10 s and 60°C for 30 s. Expression of β-actin was used as an endogenous control. Relative gene expression was calculated using 2^-ΔΔCt^ method. The real-time PCR primer sequences are shown in Supplementary Table S1.

### Western Blot

Radio immunoprecipitation assay (RIPA) lysis buffer (Boster, Wuhan, China) was used to extract protein from spinal cord tissues (L5). BCA Protein assay kit (Thermo Scientific, Waltham, MA, United States) was used to measure protein concentrations. Proteins (60 μg) were separated by 10% SDS/PAG. The membranes were blocked by 5% non-fat milk for 30 min at room temperature and then immunoblotted with the following primary antibodies: CDK6 antibody (cat no. 13331, Rabbit monoclonal, diluted at 1:1000), DNMT1 antibody (cat no. 5032, Rabbit monoclonal, diluted at 1:1000), DNMT3A antibody (cat no. 32578, Rabbit monoclonal, diluted at 1:1000), DNMT3B antibody (cat no. 57868, Rabbit monoclonal, diluted at 1:1000), GAPDH antibody (cat no. 5174, Rabbit monoclonal, diluted at 1:1000). All antibodies were purchased from Cell Signaling Technology. Membranes were then incubated with peroxidase-conjugated secondary antibody, and specific bands were detected with a Bio-Rad (Hercules, CA, United States) imaging system.

### Immunofluorescence Assays

Spinal cord tissues were cut into twenty-μm thick sections for immunofluorescence assays. After permeabilized with 0.5% Triton-100, the sections were blocked with BSA for 60 min at 37°C. The sections then were incubated with primary antibody (cat no. ab68428, anti-GFAP, 1:500; cat no. ab15690, anti-Iba-1, 1:200, Abcam, Cambridge, United Kingdom) at 4°C overnight. After that, sections were washed by PBS and secondary antibody was added to incubation at 37°C for 1 h. The coverslips were stained with DAPI (1:1000, Santa Cruz Biotechnology) for 2 min at room temperature and mounted. Fluorescence microscope (Nikon ECLIPSE 80i, Nikon Corporation, Tokyo, Japan) was used to immunofluorescence acquire images. ImageJ (version 1.8) was used to quantify the fluorescence intensity on 8 sections per animal at 40× magnification.

### Proteomics Analysis

Total proteins were extracted and purified from fresh spinal cord tissues (L5) of Lv-PKIA-AS1 infected mice and the control mice using a ReadyPrep^TM^ Protein Extraction kit (Bioscience) following the procedure recommended by the manufacturer. The proteomics analysis was performed as previously described (Yang et al., 2014). Protein samples were separated by two-dimensional polyacrylamide gel electrophoresis (2-DE). Samples containing 150 μg of protein were diluted to 450 μl with rehydration solution and used for isoelectric focusing. The proteins were electrophoresed in SDS–PAGE and then stained with coomassie brilliant blue dye. Spot-detect and determine the quantity were analyzed by DeCyder software version 6.5 (GE). The statistical significance was assessed by using a one-way ANOVA analysis. Protein spots were selected as the mean ratio was greater than 1.5-fold or less than -1.5-fold.

### Cell Culture and Cell Infection

PC12 cells were purchased from the American Type Culture Collection (Manassas, VA, United States). The cells were cultured in DMEM medium (Gibco) supplemented with 10% fetal bovine serum (Invitrogen, Carlsbad, CA, United States) and 1% penicillin/streptomycin in a 5% CO2 incubator at 37°C.

The cells were infected with the lentiviral transduction particles for PKIA-AS1. Briefly, when the cells confluence reached 80–90% in 6-well plates, the cells were infected with lentivirus [multiplicity of infection (MOI) = 50] in the presence of 5 mg/ml polybrene (Sigma-Aldrich, St. Louis, MO, United States) for 48 h.

### Luciferase Reporter Assay

To test the promoter activity, CDK6 promoter was cloned into the pGL3-Basic vector using the Fast-Fusion^TM^ Cloning Kit (FulenGen, Guangzhou, China). Luciferase reporter constructs were co-transfected with pRL-TK (Promega) into PC12 cells, followed by infection with Lv-PKIA-AS1. Dual Luciferase Reporter Assay Kit (Promega) was used to measure the luciferase activity according to the manufacturer’s instruction.

### RNA-Binding Protein Immunoprecipitation Assay

RNA-binding protein immunoprecipitation assay was conducted using Magna RIP Kit (EMD Millipore, Billerica, MA, United States) according to the manufacturer’s instruction. After the cells were lysed by RIP lysis buffer, magnetic beads conjugated to human anti-Ago2 antibody (Millipore) or control antibody (normal mouse IgG; Millipore) were added into cell lysate for incubation at 4°C overnight. CDK6 and PKIA-AS1 expression were measured by qRT-PCR.

### Quantitative Methylation-Specific PCR

CDK6 promoter (-2000–+150) methylation status was measured by MSP. Genomic DNA (1 μg per sample) extracted by the Qiagen FFPE DNA Kit (Qiagen, CA, United States) was modified with bisulfite using the EZ DNA Methylation-Gold Kit (Zymo, Orange County, CA, United States) according to the manufacturer’s instructions. Bisulfate-treated DNA was used to perform quantitative methylation-specific PCR (MSP). The qPCR steps were as following: initial denaturation at 95.0°C for 3 min; 39 cycles of 95.0°C for 10 s and 60°C for 30 s. The primers were as following: methylated-specific primer, forward, 5′-AGGCGGTTGTAGTTTTTGTAGTC-3′, reverse, 5′-ATTATTATTATTACTTTCCCACGCT-3′; unmethylated-specific primer, forward, 5′-GGAGGTGGTTGTAGTTTTTGTAGTT-3′, reverse, 5′-ATTATTATTATTACTTTCCCACACT-3′.

### ELISA

Rat IL-1β, IL-6, IL-12, and TNFα ELISA kit was purchased from Cwbiotech (Beijing, China). The lumbar spinal cord segments (L5) were removed and were homogenized in a lysis buffer. ELISA was performed according to manufacturer’s protocol.

### Statistical Analysis

The experiments repeated at least three times. Data are expressed as means ± SEM. Statistical analyses were processed on GraphPad Prism software (GraphPad Software Inc., La Jolla, CA, United States). Student’s *t*-test and one-way ANOVA with Bonferroni test were used to assess the statistical significance of the differences between two groups and multiple groups, respectively. Two-way repeated measure ANOVA with Bonferroni post-test for multiple comparisons at each time point was used to analyze PWT and PWL. P less than 0.05 was considered statistically significant.

## Results

### Identification of Differentially Expressed lncRNAs in a Rat Spinal Nerve Ligation (SNL) Model of NP

**Figure 1:**
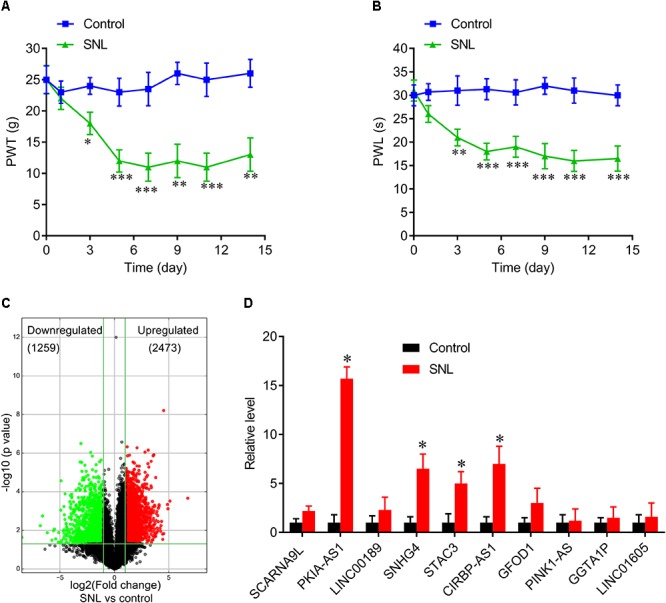
Differentially expressed lncRNAs in spinal cord of SNL rats. **(A,B)** Spinal never ligation induced mechanical hyperalgesia **(A)** and thermal hyperalgesia **(B)** in rats. *N* = 8 for each group, ^∗^*P* < 0.05, ^∗∗^*P* < 0.01, ^∗∗∗^*P* < 0.001 vs. control group. **(C)** Volcano plot shown the downregulated and upregulated lncRNAs. **(D)** Real-time PCR data confirmed the expressions of top 10 upregulated lncRNAs in spinal cord of SNL rats and control group. ^∗^*P* < 0.05 vs. control group. SNL, spinal never ligation; PWT, paw withdrawal threshold; PWL, paw withdrawal latency; lncRNA, long non-coding RNA.

To investigate the role of lncRNAs in neuropathic pain induced by spinal cord injury, we established a SNL rat model (Figure 1A,B). Total RNA was extracted from spinal cord tissues of these SNL rats and sham-operated control rats to perform lncRNA array analysis. We successfully identified 1259 downregulated lncRNAs and 2473 upregulated lncRNAs in SNL rats compared to controls (Figure 1C). Table 1 presents the detail information on the top 10 most upregulated and top 10 most downregulated lncRNAs. We then investigated the profiles of the 10 most highly upregulated lncRNAs in the spinal cord of SNL rats by real-time quantitative PCR. These substantially upregulated lncRNAs, included PKIA-AS1 (fold change >15), SNHG4 (fold change >5), STAC3 (fold change >5), and CIRBP-AS1 (fold change >5) (Figure 1D). Specific overexpression in the SNL model strongly suggests that these differentially expressed lncRNAs are involve in the pathogenesis of neuropathic pain. In this study, we focused on the contributions of PKIA-AS1 to SNL-associated NP development as it exhibited the greatest upregulation.

**Table 1:** The detail information of the top 10 up-regulated and 10 down-regulated lncRNAs.

Upregulated lncRNAs	Fold changes (SNL/control)	*P*-value	Downregulated lncRNAs	Fold changes (SNL/control)	*P*-value
SCARN9L	29.5890184	0.00520449	CTA-363E6.5	-20.526302	0.00627821
PKIA-AS1	28.6970089	0.00230417	MIR99AHG	-13.0203657	0.03423339
LINC00189	9.4945753	0.00784969	LINC01168	-10.8343423	0.00972820
SNHG4	8.536707	0.00093759	TAPT1-AS1	-10.3589502	0.03339935
STAC3	7.0920605	0.01445976	LOC10192705	-9.7866859	0.03897605
CIRBP-AS1	6.8681445	0.02234753	HMGA1P7	-8.943874	0.00490554
GFOD1	6.7029805	0.01379864	SATB2-AS1	-8.616979	0.00299287
PINK1-AS	6.5001638	0.0191133	SIGLEC17P	-8.3775419	4.70013E-05
GGTA1P	5.7075659	0.00586269	MEF2C-AS1	-7.6937744	0.01728860
LINC01605	5.2351126	0.00020316	LANCL1-AS1	-6.4846909	0.01137424

### Effects of PKIA-AS1 on Spinal Cord Ligation-Induced Neuropathic Pain

**Figure 2:**
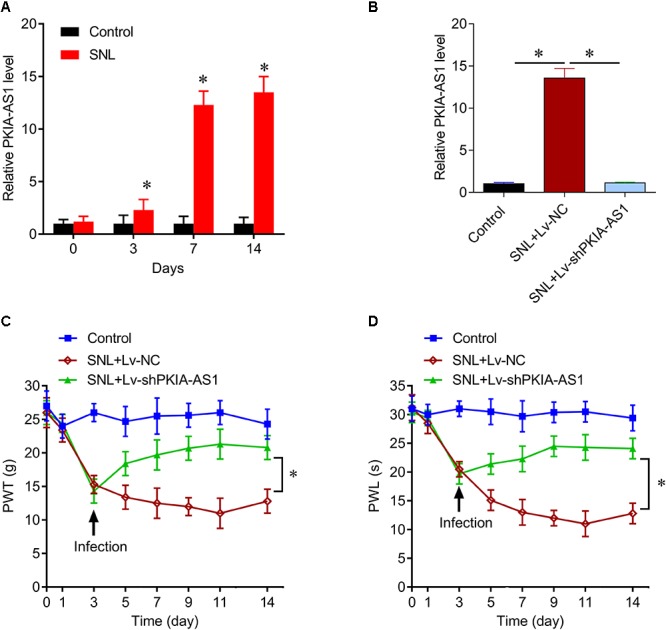
lncRNA PKIA-AS1 expression is increased in the SNL spinal cord and contributes to the SNL-induced neuropathic pain. **(A)** Real-time PCR data show the expressions of PKIA-AS1 in the SNL spinal cord on different time points. **(B)** Real-time PCR data show that the expressions of PKIA-AS1 in the SNL spinal cord treated with shPKIA-AS1 lentivirus were significantly lower than that of the SNL+Lv-NC group. **(C,D)** shRNA silencing of PKIA-AS1 alleviates mechanical hyperalgesia **(C)** and thermal hyperalgesia **(D)** in the SNL rats. *N* = 8 for each group, ^∗^*P* < 0.05 vs. control group. SNL, spinal never ligation; PWT, paw withdrawal threshold; PWL, paw withdrawal latency; lncRNA, long non-coding RNA.

Similar to NP development, expression of PKIA-AS1 increased over time following SNL (Figure 2A). Therefore, we investigated whether reducing PKIA-AS1 expression in SNL rats by shRNA knockdown can attenuate SNL induced neuropathic pain. Real-time PCR demonstrated that PKIA-AS1 expression in spinal cord of SNL rats was markedly downregulated 3 days following infection with a lentivirus vector targeting PKIA-AS1 (Lv-PKIA-AS1) compared with the SNL rats infected with control lentivirus (*P* < 0.05, *N* = 5; Figure 2B).

The effects of PKIA-AS1 shRNA on both mechanical and thermal hyperalgesia were tested by measuring the paw withdrawal threshold (PWT) and latency (PWL). Animals developed progressive mechanical hyperalgesia following SNL as evidenced by reduced PWT that plateaued on the 7th day after surgery. At 3 days post-surgery, PWT was significantly lower in rats earmarked for Lv-NC and Lv-shPKIA-AS1 infection (SNL+Lv-NC and SNL+Lv-shPKIA-AS1 groups, respectively) compared to sham-operated controls. The PWT of the SNL+Lv-shPKIA-AS1 group rose significantly above that in the SNL+Lv-NC group following infection (*P* < 0.05, *N* = 8 rats per group), indicating reduced hyperalgesia. However, the PWT of the SNL+ Lv-shPKIA-AS1 group remained slightly lower than that of the control group (Figure 2C). Similarly, the PWL was significantly shorter in the SNL+Lv-NC, and SNL+ Lv-shPKIA-AS1 groups compared with the control group on the 3th day after surgery, but was significantly higher in the SNL+Lv-shPKIA-AS1 group than the SNL+Lv-NC group following infection (*P* < 0.05, *N* = 8 rats per group) (Figure 2D). These results suggest that knockdown of PKIA-AS1 attenuates SNL-induced neuropathic pain.

**Figure 3:**
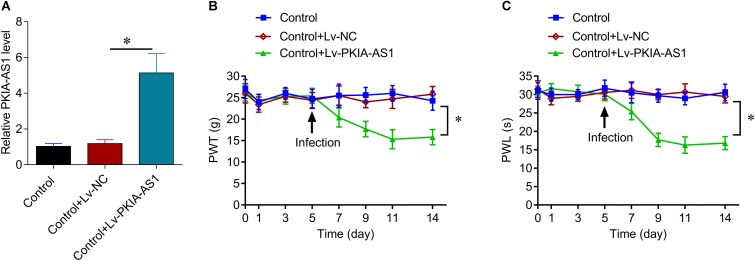
Overexpression of PKIA-AS1 is sufficient to cause pain behavior in rats. **(A)** Real-time PCR show that the expression of PKIA-AS1 in the control rats 6 days after infection of PKIA-AS1 lentivirus was significantly higher than that of the control group. ^∗^*P* < 0.05. **(B,C)** Overexpression of PKIA-AS1 produced mechanical hyperalgesia **(B)** and thermal hyperalgesia **(C)** in the control rats. *N* = 8 for each group, ^∗^*P* < 0.05. PWT, paw withdrawal threshold; PWL, paw withdrawal latency.

Next, we assessed whether PKIA-AS1 overexpression alone is sufficient to cause neuropathic pain–like symptoms in uninjured control rats. Seven days after infection with Lv-PKIA-AS1, real-time PCR showed that expression of PKIA-AS1 in the spinal cord was fivefold higher than in control rats or rats infected with the control lentivirus (*P* < 0.05, *N* = 5) (Figure 3A). The PWT in rats infected with Lv-PKIA-AS1 started to decrease by the second day post-infection and was even lower on days 4, 6, and 9 (*P* < 0.01, *N* = 8 rats per group) compared with rats infected with control lentivirus (Figure 3B), indicating progressive development of hyperalgesia. Similarly, PWL was significantly shorter in rats following Lv-PKIA-AS1 infection compared to rats infected with the control lentivirus (*P* < 0.05, *N* = 8 rats for each group) 2, 4, 6, and 9 days after infection (Figure 3C). These results suggest that overexpression of PKIA-AS1 alone is sufficient to produce hyperalgesia.

### PKIA-AS1-Induced Neuropathic Pain Is Associated With Neuroinflammation

**Figure 4:**
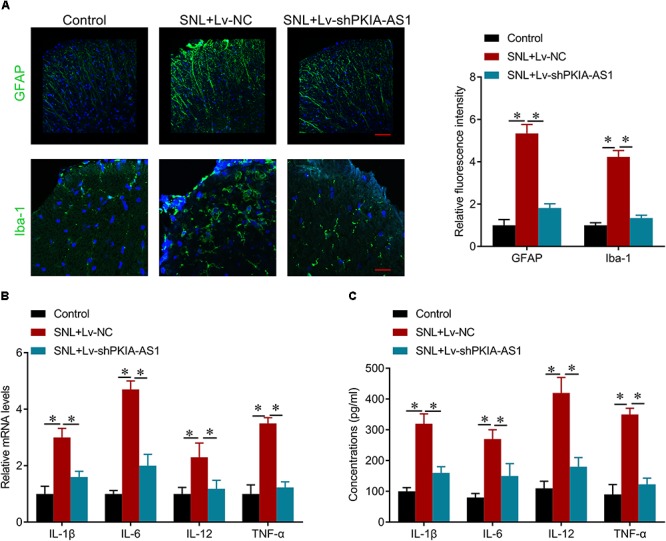
shRNA silencing of PKIA-AS1 repressed neuroinflammation in SNL rats. **(A)** Immunofluorescence assay results show that silencing of PKIA-AS1 repressed SNL-mediated astrocyte and microglia activation evaluated by GFAP and Iba-1, respectively. Scale bar: 100 μm, *N* = 8 for each group. **(B)** mRNA levels of IL-1β, IL-6, IL-12, and TNF-α in L5 spinal cord tissues in the rats 6 days after infection of PKIA-AS1 lentivirus. **(C)** The protein levels of IL-1β, IL-6, IL-12, and TNF-α in the L5 spinal cord of rats were tested by ELISA at postoperative day 6. *N* = 8 for each group; ^∗^*P* < 0.05.

To investigate the effects of PKIA-AS1 on neuroinflammation associated with NP, we evaluated the activation of astrocyte and microglia as well as the expression levels of inflammatory cytokines interleukin (IL)-6, IL-1β, IL-12 and tumor necrosis factor (TNF-)-α expression in spinal cord. As expected, SNL induced astrocytic and microglial activation as evaluated by increased GFAP and Iba-1 expression levels, respectively, while PKIA-AS1 knockdown substantially reduced glial activation (Figure 4A). PKIA-AS1 knockdown also inhibited SNL-mediated upregulation of IL-6, IL-1β, and TNF-α at both mRNA and protein levels in spinal cord (Figure 4B,C). These findings suggest that knockdown of PKIA-AS1 represses neuropathic pain by inhibiting neuroinflammation.

### PKIA-AS1 Interacts With CDK6

**Figure 5:**
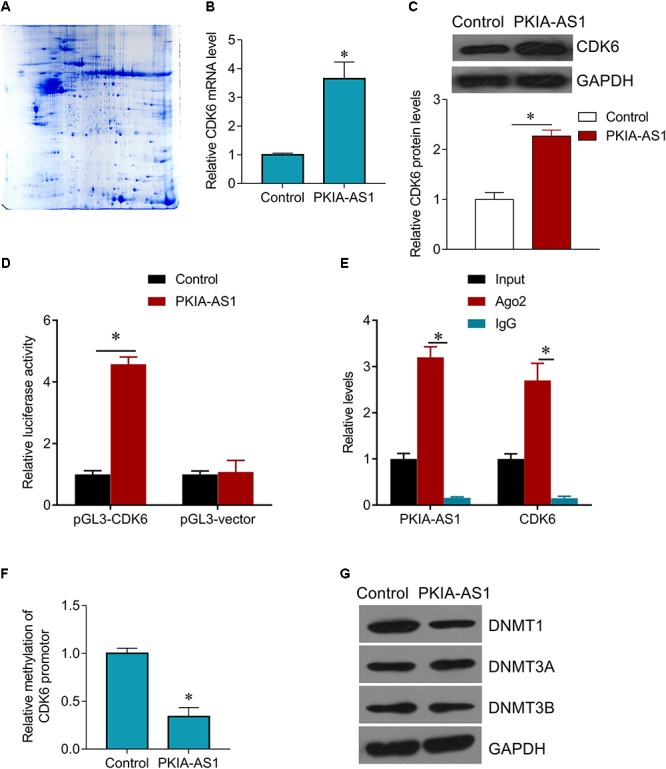
PKIA-AS1 epigenetically regulates CDK6 by hypomethylating its promotor. **(A)** The differential protein spots from 2-DE gels by differential proteomics analysis. The gel was stained with coomassie brilliant blue. 2-DE, two-dimensional polyacrylamide gel electrophoresis. **(B)** CDK6 mRNA expression in PC12 cells infected with Lv-PKIA-AS1. **(C)** CDK6 protein expression in PC12 cells infected with Lv-PKIA-AS1. **(D)** Dual luciferase assay shown that PKIA-AS1 induced CDK6 promotor activity. **(E)** The correlation between PKIA-AS1, CDK6 promotor and Ago2 was detected by RIP assay. Cellular lysates were immunoprecipitated using Ago2 antibody or IgG. PKIA-AS1 and CDK6 promotor expression was tested by qRT-PCR. **(F)** PC12 cells were infected with PKIA-AS1 lentivirus. qMSP was perform to analyze CDK6 promoter methylation. **(G)** DNMT1, DNMT3a, and DNMT3b protein levels were assessed by western blot in PC12 cells infected with Lv-PKIA-AS1; ^∗^*P* < 0.05.

The potential mechanisms underlying regulation of neuropathic pain by PKIA-AS1 were then investigated. To identify possible targets of PKIA-AS1, we screened numerous differentially expressed proteins in spinal cord tissues after PKIA-AS1 overexpression (Figure 5A). The detail information on the top 10 most upregulated and downregulated proteins is listed in Table 2. One of the most upregulated proteins is CDK6, which has been strongly implicated in inflammation (Handschick et al., 2014). We then confirmed the expression of CDK6 in spinal cord tissues from rats infected with PKIA-AS1. Indeed, Lv-PKIA-AS1 infection induced CDK6 overexpression at both mRNA and protein levels (Figure 5B,C). We further demonstrated that Lv-PKIA-AS1 infection upregulated CDK6 gene promotor activity (Figure 5D). We next tested whether PKIA-AS1 interacts with CDK6 promoter by conducting RNA pulldown assays in PC12 cells infected with Lv-PKIA-AS1 or control lentivirus. Both PKIA-AS1 and CDK6 promoter were more abundant in the Ago2 pellet than in the IgG pellet (Figure 5E), demonstrating that the CDK6 promoter interacts PKIA-AS1. We further tested how PKIA-AS1 regulates CDK6 promoter activity. The methylation status of CDK6 promoter was evaluated by quantitative methylation-specific PCR (qMSP) of the PC12 cells following Lv-PKIA-AS1 infection. Overexpression of PKIA-AS1 downregulated methylation of CDK6 promoter, suggesting that enhanced CDK6 expression in the SNL spinal cord is mediated by promoter hypomethylation induced by PKIA-AS1 (Figure 5F). In addition, PKIA-AS1 overexpression significantly inhibited DNMT1 expression, but not DNMT3A and DNMT3B expression (Figure 5G), suggesting that PKIA-AS1 regulates CDK6 expression by decreasing DNMT1-catalyzed methylation of CDK6 promoter.

**Table 2:** The detail information of the top 10 most up-regulated and 10 most down-regulated proteins.

Upregulated proteins	Fold changes (PKIA-AS1/control)	*P*-value	Downregulated proteins	Fold changes (PKIA-AS1/control)	*P*-value
CDK6	19.33	0.000730853	Galectin-7	-14.06	0.005513955
Myosin	14.13	0.002566788	Histidine triad nucleotide-binding protein 2	-12.01	0.004340288
Alpha-crystallin B chain	10.45	0.000169286	Palate lung and nasal epithelium clone protein	-10.46	0.025461992
Myoglobin	9.33	0.017749647	Apolipoprotein A	-9.32	0.006539903
Creatine kinase M-type	9.91	0.007387786	Pyridoxine-5′-phosphate oxidase	-8.33	0.005393046
Creatine kinase M-type	8.90	0.001036429	Peroxiredoxin-2	-7.58	0.000836037
Fibrinogen	7.25	0.001054321	Fructose-bisphosphate aldolase A	-6.64	0.010053958
Pyruvate kinase	6.80	0.001121332	Ribosomal protein SA	-4.41	0.036115645
Model of actin-fimbrin abd2 complex	5.00	0.001233447	Keratin, type II cytoskeletal 1	-3.78	0.016436261
Vimentin	5.23	0.00020316	Keratin 5	-6.48	0.01137424

### PKIA-AS1 Regulates Neuropathic Pain Progression Through CDK6

**Figure 6:**
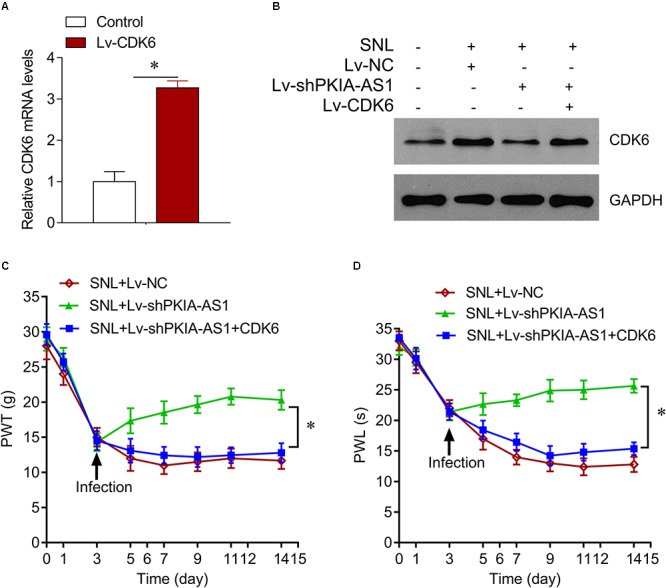
PKIA-AS1 modulated neuropathic pain by regulating CDK6. **(A)** CDK6 mRNA expression in SNL rat models infected with Lv-CDK6 6 days after lentivirus infection. **(B)** CDK6 protein expression in the spinal cord tissues of rats infected with indicated viruses. **(C)** The effect of CDK6 on mechanical allodynia was evaluated by PWT. **(D)** The effect of CDK6 on thermal hyperalgesia was assessed by PWL. *N* = 8 for each group; ^∗^*P* < 0.05. PWT, paw withdrawal threshold; PWL, paw withdrawal latency.

**Figure 7:**
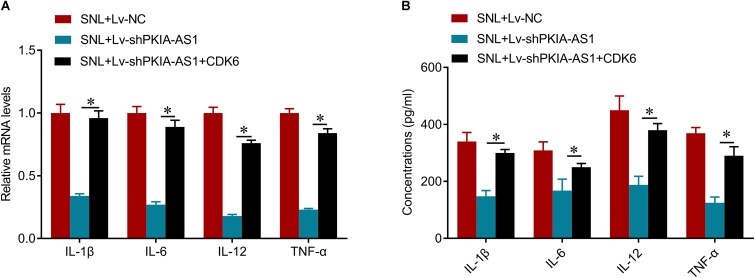
shRNA silencing of PKIA-AS1 inhibits neuroinflammation by regulating CDK6 in SNL rats. **(A)** mRNA levels of IL-1β, IL-6, IL-12, and TNF-α in L5 spinal cord tissues in the rats 6 days after lentivirus infection. **(B)** The protein levels of IL-1β, IL-6, IL-12, and TNF-α in the L5 spinal cord of rats were tested by ELISA 6 days after lentivirus infection. *N* = 8 for each group; ^∗^*P* < 0.05.

We further investigated whether PKIA-AS1 controls neuropathic pain via CDK6. Expression of CDK6 was significantly increased in SNL model rats compared with control rats, whereas CDK6 expression was inhibited by PKIA-AS1 knockdown. In addition, PKIA-AS1 knockdown-mediated inhibition of CDK6 was reversed by Lv-CDK6 infection (Figure 6A,B). We found that Lv-CDK6 infection reversed the attenuation of mechanical allodynia and thermal hyperalgesia by Lv-shPKIA-AS1 infection following SNL (Figure 6C,D). Moreover, Lv-CDK6 reversed the suppression of IL-1β, IL-6, IL-12, and TNF-α mRNA and protein expression by Lv-shPKIA-AS1 (Figure 7A,B). Collectively, these results indicate that an epigenetic regulatory pathway involving PKIA-AS1 and CDK6 mediates SNL-induced neuropathic pain.

## Discussion

We have identified the lncRNA PKIA-AS1 as a major regulator of SNL associated with NP. First, PKIA-AS1 was significantly upregulated in the SNL model rat spinal cord. Furthermore, both gain-of-function and loss-of-function studies confirmed that PKIA-AS1 was necessary for generating SNL-induced neuropathic pain. Our results suggest that PKIA-AS1 is a critical regulator of SNL-induced neuropathic pain, and that manipulation of PKIA-AS1 expression could potentially be used to improve spinal nerve ligation-induced neuropathic pain.

Peripheral inflammation and nerve injury can alter the expression of numerous genes, including lncRNAs, in affected pain pathways (Jiang et al., 2015; Wu et al., 2018). Moreover, numerous lncRNAs have been identified in the pain-related regions, such as dorsal root ganglion and spinal cord of mouse, rat, and human (Hu et al., 2016; Dou et al., 2017; Mo et al., 2018). The spinal cord dorsal horn is responsible for relaying and modulating pain-related signals from nociceptors to the supraspinal brain regions. The expression of the lncRNA colon cancer associated transcript-1 (CCAT1) was found to decrease in the spinal cord, dorsal root ganglion, hippocampus, and anterior cingulate cortex from day 1 to day 5 after chronic sciatic nerve injury (CCI). Over-expression of lncRNA CCAT1 alleviated CCI-induced mechanical allodynia (Dou et al., 2017). In the present study, the expression of another lncRNA, PKIA-AS1 was markedly increased in the spinal cord following SNL and was associated with heightened pain sensitivity, whereas shRNA-mediated knockdown of PKIA-AS1 alleviated thermal and mechanical hypersensitivity caused by SNL. These results suggest that upregulation of PKIA-AS1 in the spinal cord may be an important contributor to SNL-induced neuropathic pain. This possibility is further supported by our findings that PKIA-AS1 overexpression in spinal cord of uninjured rats was sufficient to induce pain-related behaviors. In the present study, the expression profiles of lncRNA were obtained from whole, unperfused spinal cord tissues after SNL so the specific cells overexpressing this lncRNA are currently unknown. Additional studies are needed to assess whether PKIA-AS1 and the other lncRNAs differentially expressed in the SNL spinal cord are altered in neurons, microglia. and (or) astrocytes.

We identified many possible targets of PKIA-AS1 regulation by proteomic assay, and confirmed that CDK6 is an interaction partner of PKIA-AS1. We further demonstrated that PKIA-AS1 interacts with CDK6 promoter and enhances promoter activity by modulating its methylation status. Our results strongly support the notion that PKIA-AS1 interacts with CDK6 promoter to enhance its expression and function. Cyclin-dependent kinases (CDKs) control diverse biological processes via the regulation of the cell cycle and gene expression (Peng et al., 2013). CDKs transcriptionally induce proinflammatory genes during the G1 phase of cell cycle. Further, cytokine-induced recruitment of CDK6 to the nuclear chromatin fraction is associated with activation of NF-κB, STAT, and AP-1 transcription factor families. CDK6 can recruit NF-κB subunit p65 to its target sites (Schmitz and Kracht, 2016). The demonstrated involvement of CDKs in proinflammatory gene expression makes these proteins promising target for treatment of chronic inflammatory diseases. CDK6 has been implicated in the proliferation, differentiation, and survival of neurons (Beukelaers et al., 2011; Mi et al., 2013; Caron et al., 2018; Hasenpusch-Theil et al., 2018). For example, preventing CDK6 over-activation can reduce 6-hydroxy-dopamine-induced neuronal death (Alquezar et al., 2015). According to previous studies, CDK6 is localized to the cytoplasm (Kohrt et al., 2009). CDK6 also regulates astrocyte proliferation and microglial activation (Zhao et al., 2013; Gu et al., 2016), suggesting that CDK6 is involved with inflammatory pain. In our study, we demonstrate for the first time that PKIA-AS1 epigenetically controls CDK6 expression by modulating promoter methylation, thereby regulating proinflammatory cytokine release, and ultimately controlling neuropathic pain.

In conclusion, PKIA-AS1 plays an important role in the pathogenesis of neuropathic pain through direct interaction with CDK6. Silencing PKIA-AS1 may be a complementary approach to reduce neuropathic pain induced by spinal nerve ligation through downregulation of CDK6 expression.

## Author Contributions

J-ZH and H-BL designed the study. JZH and Z-JR performed cell biological experiments and *in vivo* experiments. ML, PL, L-YJ, Z-XL, C-YD and YC analyzed and interpreted the data. All authors contributed to writing the manuscript. All authors read and approved the final manuscript.

## Conflict of Interest Statement

The authors declare that the research was conducted in the absence of any commercial or financial relationships that could be construed as a potential conflict of interest.
