# Supplementary material for: Silencing of lncRNA PKIA-AS1 Attenuates Spinal Nerve Ligation-Induced Neuropathic Pain Through Epigenetic Downregulation of CDK6 Expression
Source: Front Cell Neurosci. 2019 Feb 20;13:50. doi: 10.3389/fncel.2019.00050 (PMC6401634; doi:10.3389/fncel.2019.00050)
Supplement: Supplementary file 1 [file Table_1.docx]

Supplemental Table 1 The primer sequences used in qPCR

| gene | Sense (5’-3’) | Anti-sense (5’-3’) |
| --- | --- | --- |
| SCARNA9L | AGAAGGCTTTCCGGTCTACCT | CCTCACCCCCAATCTCATTCA |
| PKIA-AS1 | TCAGCAAGGGCAAATCCCAA | GCAATGCCAAAGGACGTTGT |
| LINC00189 | GATGCAATTCTGGCCTTGGAG | TCATTGACCAAGAGCCCCTTC |
| SNHG4 | AGTAGGGCATCCTTCACCCA | TTAAGTCCCCTACCCCCATCT |
| STAC3 | CCGAACCTACAGCGGCTAAA | GTGGGGCTTATCGTTGACCA |
| CIRBP-AS1 | CTCTATGGCCACCACTTGCT | GGGATTTCCCTGGGATGAGC |
| GFOD1 | GTGTCGGGTTTTCTTGGGTG | GTCGCAGCTCCAGTTGTACT |
| PINK1-AS | CAGGCAGCAGCCAAAATCTG | GGCCCCAGTGAAGTATCGTG |
| GGTA1P | TGACAGCAGTGCTCAGAAGG | AATCTCGCAGTCCCAACAGG |
| LINC01605 | GCACTGAACCCCTTGTCTGA | AAGACTCCGTTCTAGGGCCA |
| CDK-6 | CCGAGTAGTGCATCGCGATCTAA | CTTTGCCTAGTTCATCGATATC |
| IL-1β | GAAATGCCACCTTTTGACAGTG | TGGATGCTCTCATCAGGACAG |
| IL-6 | CTCTGGCTTTGTCTTTCTTGTTATCTTT | AGTTGTGCAATGGCAATTCTGA |
| IL-12 | AGACCCTGCCCATTGAACTG | CCTTTGCATTGGACTTCGGC |
| TNF-α | AGGCGGTGCCTATGTCTCAG | GCTCCTCCACTTGGTGGTTT |
| β-actin | GCCCTATAAAACCCAGCGGC | TCGATGGGGTACTTCAGGGT |
